# STAT3, a Key Parameter of Cytokine-Driven Tissue Protection during Sterile Inflammation – the Case of Experimental Acetaminophen (Paracetamol)-Induced Liver Damage

**DOI:** 10.3389/fimmu.2016.00163

**Published:** 2016-05-02

**Authors:** Heiko Mühl

**Affiliations:** ^1^Pharmazentrum Frankfurt/ZAFES, University Hospital Goethe-University Frankfurt am Main, Frankfurt am Main, Germany

**Keywords:** acetaminophen, acute liver injury, hepatocytes, STAT3, IL-6, IL-11, IL-13, IL-22

## Abstract

Acetaminophen (APAP, *N*-acetyl-*p*-aminophenol, or paracetamol) overdosing is a prevalent cause of acute liver injury. While clinical disease is initiated by overt parenchymal hepatocyte necrosis in response to the analgetic, course of intoxication is substantially influenced by associated activation of innate immunity. This process is supposed to be set in motion by release of danger-associated molecular patterns (DAMPs) from dying hepatocytes and is accompanied by an inflammatory cytokine response. Murine models of APAP-induced liver injury emphasize the complex role that DAMPs and cytokines play in promoting either hepatic pathogenesis or resolution and recovery from intoxication. Whereas the function of key inflammatory cytokines is controversially discussed, a subclass of specific cytokines capable of efficiently activating the hepatocyte signal transducer and activator of transcription (STAT)-3 pathway stands out as being consistently protective in murine models of APAP intoxication. Those include foremost interleukin (IL)-6, IL-11, IL-13, and IL-22. Above all, activation of STAT3 under the influence of these cytokines has the capability to drive hepatocyte compensatory proliferation, a key principle of the regenerating liver. Herein, the role of these specific cytokines during experimental APAP-induced liver injury is highlighted and discussed in a broader perspective. In hard-to-treat or at-risk patients, standard therapy may fail and APAP intoxication can proceed toward a fatal condition. Focused administration of recombinant STAT3-activating cytokines may evolve as novel therapeutic approach under those ill-fated conditions.

## Introduction

Acute liver injury (ALI) is a major burden of health care systems worldwide. Viral infections and side effects of pharmacotherapy stand out among pathological challenges provoking ALI. Specifically, overdosing of the weak-to-moderate analgesic acetaminophen (APAP; *N*-acetyl-*p*-aminophenol; or paracetamol) is regarded as one major cause of ALI in the developed countries. Notably, over-the-counter availability, underrated toxicity, and a narrow therapeutic margin further APAP misuse/intoxication which, if not timely treated with its antidote *N*-acetylcysteine, can proceed toward a fulminant condition requiring transplantation for patient survival (1–3).

Specifically, APAP is hold responsible for up to 80,000 emergency visits, 2500 hospitalizations and 500 fatal intoxications in the United States annually (2, 4). A recent study analyzing between 2005 and 2007 patients from selected European countries documented 114 drug overdose-related cases of ALI demanding transplantation (of 600 totals). Ninety-seven percent (111 cases) of those concerned APAP (5). In Germany, 850 hospitalizations due to APAP intake were recorded 2012 for patients with statutory health insurance. However, only four fatalities were documented (6). Altogether, epidemiological studies indicate noticeable variations in the incidence of severe APAP-induced ALI in different populations within Europe (5) and the developed countries altogether.

On a cellular level, liver injury by APAP is regarded a two-hit process involving initial direct induction of hepatocyte cell death and, subsequent to that, activation of innate immunity that triggers an inflammatory response having the complex potential to either aggravate disease or to actually support tissue repair and hepatic regeneration (7–12).

Hepatocyte cell death, being at the root of APAP toxicity, is dependent on drug metabolizing cytochrome P450 enzymes (Cyp), particularly Cyp2e1 and Cyp1a2 (8, 13). These enzymes generate from APAP poisonous *N*-acetyl-*p*-benzoquinone imine (NAPQI), a highly reactive metabolite capable of coupling to protein sulfhydryl groups thereby disturbing hepatocyte cell physiology. Specifically, NAPQI mediates mitochondrial oxidative stress, drop in ATP generation, c-jun N-terminal kinase (JNK) activation, and eventually cell death (8, 14). Although apoptosis and necrosis as well as necrosis-related necroptosis have all been detected during experimental APAP-induced ALI, the latter two types of cell demise prevail in the context of pathological intoxication. Notably, as opposed to immune-deactivating apoptosis, necrosis and necroptosis connect to activation of innate immunity and inflammation (7, 8, 14, 15) whereby the leukocytic cell compartment becomes involved into course and outcome of APAP-induced ALI.

## The Complex Role of Innate Immunity and Inflammatory Cytokines in Experimental Murine APAP-Induced Liver Injury

Key to sterile necro-inflammation, as detected in APAP-induced ALI, is release of danger-associated molecular patterns (DAMPs) from cells undergoing necrosis. Once increasingly present in the extracellular compartment or later on taken up by leukocytes, those are detected by sensors of innate immunity, e.g., toll-like receptors (TLR), setting in motion inflammatory processes that can drive pathology but also setting the stage for parenchymal tissue repair and regeneration (9–12, 16, 17).

DAMPs reported to mediate pathological immunoactivation during APAP-induced ALI include high-mobility group box 1 protein (HMGB1) (18–20) and histones (21). Both couple to TLR4 on hepatic monocytes/macrophages, including resident Kupffer cells. Besides that, HMGB1 was found to activate the receptor for advanced glycation end product (RAGE) on neutrophils, whereas histones may mediate pathological effects also *via* TLR2. Nucleic acids released from necrotic hepatocytes likewise display a strong potential to aggravate APAP intoxication by action on TLR sensors. Specifically, DNA targeting TLR9 (22, 23) and RNA targeting leukocytic or hepatocyte TLR3 (24) contribute to hepatic injury. A pivotal role for TLR9 was confirmed by pharmacological application of a small-molecule TLR9 antagonist to mice undergoing APAP intoxication (25). Among DAMPs sensed independently from the TLR system, ATP and uric acid stand out. Both can aggravate APAP-induced ALI (26–28) supposedly by action on the inflammasome, a multiprotein complex consisting of interleukin (IL)-1β/IL-18-activating caspase-1. In this scenario, ATP binds to purinergic P2X_7_ receptors on monocytes/macrophages (including Kupffer cells) at the hepatic microenvironment connecting to cellular K^+^-efflux and subsequent inflammasome activation. After being released by dying cells or derived from degradation of nucleic acids, uric acid, on the other hand, is taken up in the form of crystals that directly activate inflammasomes and, thus, IL-1β/IL-18-dependent inflammation (29, 30).

Although, at first sight, it appears obvious that innate immunity and sterile inflammation amplify pathogenesis of APAP-induced ALI, this topic in fact is controversially discussed. For example, while several studies indicate a pathological role of TLR4 (21, 31, 32), a recent report did not endorse a disease-promoting but rather a protective function of myeloid TLR4 signaling in APAP-related liver damage. Interestingly, deleterious action of RAGE and TLR9 was confirmed in this same study (33). Another recent report disputed a pathogenic role for P2X_7_ receptors in APAP intoxication (34). While some parameters, such as mice characteristics, including their microbiome (35), or APAP dosage cannot be fully ruled out as sources of discrepancies, those divergent observations may actually reflect janus-faced functions of innate immunity and sterile inflammation in APAP-induced ALI – aggravating tissue damage, likely at an early phase of disease, but simultaneously displaying the strong potential to initiate and perpetuate hepatic repair and regeneration (36). The unique ability of the liver to, upon injury, most efficiently initiate processes aiming at preservation of organ function is driven by initial hepatocyte hypertrophy (increase in size) and an adjacent proliferative phase enabling compensatory hyperplasia (37, 38). Notably, if hepatic damage stays below a pathological threshold, the regenerative capacity of the liver can fully restore organ function in response to APAP (39, 40).

The remarkable fact of quite divergent observations concerning the role of sterile inflammation in APAP-induced ALI particularly applies to the function of nuclear factor-κB (NF-κB)-activating inflammatory cytokines that are induced adjacent–distal to innate sensing. This specifically holds true for prototypic IL-1 and tumor necrosis factor (TNF)-α (41), both produced during APAP-induced ALI (22, 24, 42–46). Whereas aggravation of disease by pretreatment of mice with recombinant TNFα is undisputed (47), modulation of endogenous TNFα biological activity, as achieved by administering neutralizing antibodies or by investigating TNF receptor-1-deficient mice, resulted in quite heterogeneous outcome. Reports, on the one hand, demonstrate amelioration of APAP-induced toxicity by application of anti-TNFα antibodies (48, 49) or by using TNF receptor-1-deficient mice (49). By contrast, other reports observed either no effect of TNFα-neutralization (50, 51) or even aggravation of disease as detected using TNF receptor-1-deficient mice (52, 53). Those latter two studies actually indicate a tissue-protective function of endogenous TNFα in APAP-induced ALI that coincides with enhanced hepatocyte proliferation and activation of the key pro-regenerative transcription factor signal transducer and activator of transcription (STAT)-3 (54). To assess the role of IL-1 in APAP-toxicity is likewise puzzling. Either pathogenic functions (22, 55), no major role (42), or protection (56) by IL-1 has been put on record. The view that inflammatory cytokines, such as IL-1 and TNFα, have the potential to actually promote liver regeneration was recently extended to the IL-1 family member IL-36γ (57, 58). In fact, administration of IL-36 receptor antagonist and thus blockage of IL-36 biological activity during APAP-induced ALI impairs recovery in the late phase of intoxication (58). Interestingly, IL-36 mediating tissue protection likewise applies to intestinal healing (59, 60).

Altogether, current data support the concept that sterile inflammation and accompanied NF-κB-activating cytokines may promote hepatic repair and regeneration particularly in the later phase of APAP toxicity thereby affecting disease outcome (12). It is tempting to speculate that secondary induction of STAT3-activating cytokines, alike IL-6 (61), by NF-κB-activating cytokines essentially contributes to the vital process of restoring liver homeostasis in response to APAP.

## STAT3 in Hepatic Repair and Regeneration

STAT-3 is a member of the STAT family of transcription factors, which exerts decisive and context-dependent functions in inflammation, tissue survival, and carcinogenesis. Those characteristically include promotion of anti-apoptosis, proliferation, and stress resistance. Efficient activation of STAT3 is achieved under the influence of specific cytokines displaying janus-kinase signaling but also by selected growth factors, among others epidermal and platelet-derived growth factor. Phosporylation at Tyr705 is regarded a hallmark of STAT3 activation that couples to protein dimerization, nuclear translocation, and regulation of gene expression (62–65). In addition, phosphorylation at Ser727 (63, 65) and/or protein acetylation (66) amplify, whereas S-nitrosylation at Cys259 (67) and/or protein sumoylation (68) curb STAT3 activity. As already alluded to, enforcing hepatocyte anti-apoptosis and proliferation is key to liver protection by STAT3. Those functions are achieved by upregulation of gene products pivotally involved in cell fate decisions, among others, B-cell lymphoma-extra large (*bclxL*), myeloid cell leukemia-1 (*mcl1*), or *survivin* mediating anti-apoptosis as well as c-myc (*myc*), cyclin B1/D1 (*ccnb1*/*ccnd1*), or cyclin-dependent kinase-2 (*cdc2*) mediating proliferation (62, 63).

The albumin-promoter was used to generate hepatocyte-specific conditional STAT3 knockout mice in order to address the role of STAT3 in this cell type. Experiments revealed that hepatocyte STAT3 is, to a substantial part, accountable for hepatocyte proliferation and liver regeneration after murine partial hepatectomy. Notably, hepatocyte c-myc expression is aberrant and its inducibility retarded in aforementioned conditional STAT3-deficient mice undergoing this procedure (69). In a study using a similar approach, hepatocyte STAT3 was functionless regarding parameters of liver injury evaluated in early APAP-induced ALI. However, analysis in that study was performed only 6 h after APAP administration and, thus, in the initial phase of intoxication (70) – leaving open the question of STAT3 functions during the later repair/regeneration phase. Notably, increased STAT3 activation in murine liver is still detectable 24 h after APAP application (71); the same holds true for expression of STAT3-activating IL-6 (72, 73).

## Tissue Protection by STAT3-Activating Cytokines as Detected in APAP-Induced ALI: IL-6, IL-11, IL-13, and IL-22 – and IL-10

Whereas the role of NF-κB-activating cytokines in APAP-induced ALI appears complex and bewildering, STAT3-activating cytokines capable of directly targeting hepatocytes must be regarded as major drivers of liver regeneration. Those include IL-6, IL-11, IL-13, and IL-22.

Interleukin-6 is the flagship of a family of cytokines operating through transmembrane gp130 as signal transducing unit thereby coupling to activation of STAT transcription factors, in case of IL-6 foremost STAT3 (61). This also applies to its cytokine sibling IL-11 (74). Both, IL-6 and IL-11, are upregulated during initial hepatocyte injury and stay elevated, along with activated STAT3 (71), in the repair/regeneration phase at 24 h after APAP administration to mice (72, 73). In fact, protection by endogenous IL-6 was observed early on. Particularly in time-wise advanced disease 24 h (73) or 48 h (75) after APAP administration, IL-6-deficient mice endure aggravated toxicity associated with low production of hepatocyte-associated proliferating cell nuclear antigen (PCNA) and weakened liver macrophage inflammatory protein-2 (MIP-2) expression (75). Both, PCNA and MIP-2 (76), are key parameters of hepatocyte proliferation under the influence of APAP. Those observations suggest impaired recovery upon lack of IL-6. As expected, treatment of IL-6 deficient mice with recombinant IL-6 attenuated retardation of repair and regeneration (75). It is noteworthy that hepatocytes are among the few non-leukocytic cell types expressing functional IL-6 receptors and, thus, allow classical IL-6 signaling. Despite this fact, recent data indicate that trans-signaling by soluble IL-6R/IL-6 complexes (61, 77) is essential for the function of this cytokine during APAP-induced ALI (78). In fact, specific blockage of IL-6 trans-signaling by sgp130Fc (77) substantially exacerbated disease (78); whereas pretreatment of mice with hyper-IL-6 (77), a recombinant agent specifically activating trans-signaling, ameliorated APAP toxicity – albeit to a more moderate degree (78).

Interleukin-11 is a further STAT3-activating member of the IL-6 family directly targeting hepatocytes (74, 79) and, in stark contrast to IL-6, is efficiently expressed by inflamed/stressed hepatocytes under the influence of APAP (71). Autocrine or paracrine action may, thus, ensure high local IL-11 bioactivity that likely feeds back on the course of APAP-induced ALI. Notably, early data already revealed amelioration of murine APAP-toxicity by recombinant human IL-11 (80). This observation has been corroborated recently. A super-active modification of human IL-11 indeed enhanced protective hepatocyte compensatory proliferation in diseased mice. In female IL-11 receptor-deficient mice (*IL11Ra*^−/−^) aggravated toxicity and diminished hepatocyte proliferation indicate a significant role for endogenous IL-11 during APAP-induced ALI. Interestingly, this observation does not apply to male *IL11Ra*^−/−^ mice that actually display compensatory augmentation of supposedly protective IL-6 (71). It should be emphasized that female mice, compared to males, generally display reduced sensitivity toward APAP that is connected to an enhanced capability in females to restore hepatocyte mitochondrial glutathione levels (81).

Interleukin-13 is renowned as key Th2 cytokine that, however, is produced by various cell types of foremost leukocytic origin. By binding to its heterodimeric IL-4Rα/IL-13Rα1 receptor complex, IL-13 activates STAT3 (along with STAT6) in even more diverse cell types (82), including murine hepatocytes (83). Elevated systemic levels of IL-13 are well-detectable at 4, 12, and 24 h after APAP administration to mice (51, 84). Notably, exacerbated disease connecting to IL-13 blockage by neutralizing antibodies or lack of bioactivity in knockout mice firmly indicates protection by this cytokine during APAP intoxication (51). Activation of hepatocyte STAT3 by IL-13 (83) suggests direct protective action during APAP-induced ALI. However, upregulation of the supposedly detrimental IL-12/IFNγ-axis (72) during intoxication in IL-13-deficient mice (51) additionally implicates macrophage-addressing immunomodulatory functions of IL-13 (85). Whether administration of surplus recombinant IL-13 can ameliorate APAP-induced ALI has, to the best of our knowledge, not been investigated.

Interleukin-22 is mainly a lymphocyte-derived member of the IL-10 cytokine family that gained significant attention due to tissue-protective properties largely mediated by STAT3 activation specifically in epithelial (-like) cells, including hepatocytes. Accordingly, IL-22 mediates favorable effects in various preclinical disease models affecting biological barriers at the lung, intestine, and liver. Notably, IL-22 generally does not activate leukocytes (86–88). A single dosage of recombinant IL-22 is actually sufficient to ameliorate APAP toxicity in mice (43, 70). Protection by IL-22 is dependent on STAT3 (70), does not involve modulation of APAP-metabolizing cytochrome P450 enzymes but is associated with increased compensatory hepatocyte proliferation (43). The role of endogenous IL-22 during APAP-induced ALI has, to the best of our knowledge, not been investigated. Recently, a functionally relevant aspect of IL-22 biology attracted attention. A potent synergism between the IFN signaling system and IL-22 concerning activation of STAT1 was identified in human colon carcinoma cells, HepG2 hepatoma cells, and primary keratinocytes on a biochemical level (89). In contrast to STAT3, STAT1 (e.g., activated by IFNγ) promotes cell death, inhibits proliferation, is generally considered pro-inflammatory (90), and pathogenic in APAP-induced ALI (72). This regulatory path has recently been extended to murine *in vivo* pathology during viral infection (91) or graft-versus-host disease (92) and may affect the function of IL-22 not only under conditions of overt IFN production but likewise in the context of typ I IFN immunotherapy (90).

Interleukin-10 is a mainly leukocyte-derived protein that drops out of the list of aforementioned STAT3-activating cytokines because it is supposed to act foremost on leukocytic cells. IL-10 serves as principal deactivator of T cells and in particular of mononuclear phagocytes thereby modulating in STAT3-dependent manner inflammatory processes (93, 94) and holding in check potentially poisonous mediators, among others inducible nitric oxide (NO) synthase (95) -derived NO (84). During APAP intoxication systemic levels and hepatic expression of IL-10 increase. Notably, IL-10 deficient mice display enhanced sensitivity to APAP-induced ALI, which is unrelated to APAP metabolism but detectable on the level of serum ALT, morphologically, and by analysis of mortality rates (84). Since STAT3 can principally drive IL-10 expression (93, 96, 97), this regulatory path may possibly contribute to tissue protection by STAT3-activating cytokines, such as IL-6. However, the therapeutic potential of surplus exogenously applied IL-10 in APAP-induced ALI seems limited as administration of the recombinant cytokine failed to protect diseased mice (50).

Although this review focuses on cytokines efficiently targeting hepatocytes, it is important to note that modulation of murine APAP-induced ALI by endogenous IL-10 (and IL-13) unequivocally indicate a pivotal function of STAT3 also in myeloid cells (monocytes/macrophages/Kupffer cells) for determining course and outcome of APAP intoxication. Besides addressing STAT3 in hepatocytes, hepatic myeloid STAT3, thus, certainly is a further promising target for development of therapeutic strategies aiming at APAP-induced ALI.

## Translational/Therapeutic Implications and Conclusions

Administering hepatocyte STAT3-activating cytokines emerges from preclinical studies as encouraging pharmacological strategy that aims at hard-to-treat patients with APAP-induced ALI. Moreover, APAP intoxication may serve as paradigm for a whole group of injury-driven acute inflammatory liver diseases independent on the nature of the initiating insult (54). To translate preclinical knowledge to clinical application is, however, in some cases advantaged in others complicated by specific properties ascribed to aforementioned cytokines.

Although IL-6 displays significant tissue-protective characteristics, administration of the recombinant cytokine to patients is hampered by its pro-inflammatory effects especially on lymphocyte biology (61). Specifically, IL-6 promotes human IL-17 production and associated Th17 differentiation (98). Notably, IL-17 is pathogenic in murine APAP-induced ALI (19). As IL-6-induced Th17 associates with compromised Treg functions (99–101) and, if applicable, pathological antibody production (102), current knowledge supports serious concerns that administration of IL-6 to patients may initiate or enhance autoimmune inflammation.

Mice undergoing APAP toxicity did not benefit from exogenously provided IL-10 (50), which may likewise apply to human intoxication. As chief deactivator of leukocytes (93, 94), recombinant IL-10 should actually interfere with desired production of potentially pro-regenerative factors. In fact, this has been demonstrated for IL-6 and TNFα production by human Kupffer cells under the influence of active TLR4 signaling (103).

Interleukin-11 and IL-22 are functionally related cytokines that efficiently activate hepatocyte STAT3 signaling and associated downstream gene expression. Both have been described to mediate tissue protection at host/environment interfaces, in particular at the digestive tract. For example, IL-11 (104, 105) and IL-22 (106, 107) display protective properties in *Citrobacter rodentium*-driven infectious as well as in trinitrobenzene sulfonic acid chemically induced experimental colitis. Accordingly, use of both cytokines is discussed, albeit with caution, for the treatment of inflammatory bowel diseases (108). Aforementioned liver protective properties of IL-11 and IL-22 are not restricted to APAP intoxication. Among others, experimental hepatic disorders mediated by reperfusion injury (109, 110) or administration of either carbon tetrachloride (111, 112) or concanavalin A (112, 113) likewise exposed beneficial effects of both cytokines. Although the role of IL-11 and IL-22 in liver repair/regeneration should primarily be mediated by STAT3, it must be stressed that activation of MAPK- and PI3K/Akt-pathways may support IL-11/IL-22 action in this context (74, 86, 114). The feasibility of recombinant IL-11 therapy for the treatment of ALI is emphasized by its relatively favorable compatibility in clinical trials (115). In fact, recombinant IL-11 has been approved for the treatment of severe thrombocytopenia by the US Food and Drug Administration (79). At dosages showing biological activity, F-652 [Generon (Shanghai) Corporation Ltd.], an IL-22-like biopharmaceutical agent consisting of a human IL-22-Fc-fusion protein (116), is likewise reported to have a good safety profile as determined in a phase I study in healthy volunteers (http://www.businesswire.com/news/home/20151123005647/en/Generon-Collaborating-Mayo-Clinic-Initiate-Phase-IIa).

Pharmacotherapy of APAP-induced ALI must obviously be successful when initiated hours after ingestion. Whereas most studies assessed prophylactic treatment, therapeutic application has been investigated in a translational setting for IL-22. Specifically, when administered 2 h after APAP together with suboptimal N-acetylcysteine dosing, recombinant IL-22 improved murine intoxication (43). Notably, IL-22 application 2 h post-APAP is after the drop of cellular glutathione as well as the onset of APAP-adduct formation and liver necrosis (43, 117). More studies on treatment timing, however, are needed before experimental models can be translated to clinical intoxication.

**Figure 1:**
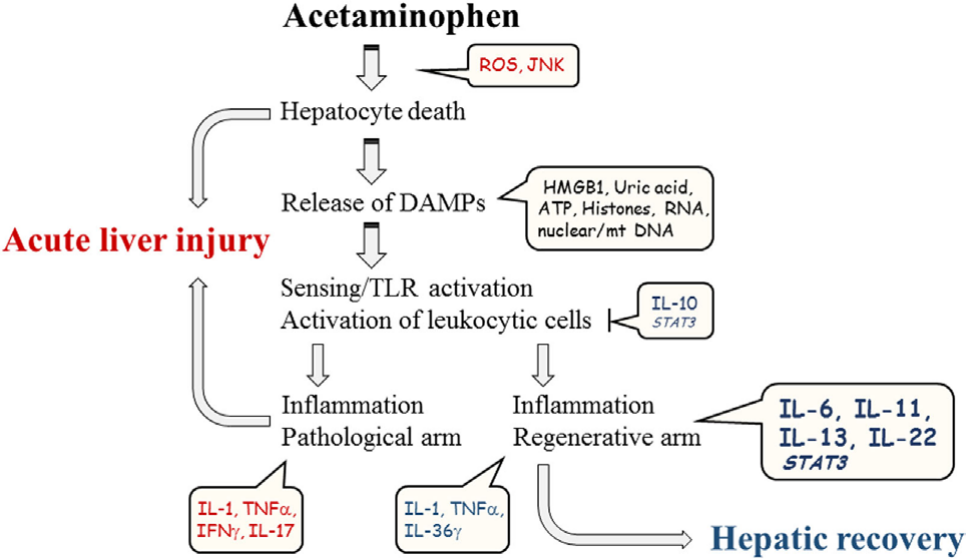
**Schematic illustration of major parameters contributing to course of experimental APAP-induced ALI**.

Altogether, APAP-induced ALI is a complex disorder determined by the extent of initial hepatotoxicity, by the nature of adjacent sterile inflammation, and by the actual regenerative potential of the liver at the time of injury (Figure 1). Preclinical data suggest that providing recombinant STAT3-activating cytokines directly targeting hepatocytes, especially IL-11 and IL-22, may evolve as additional novel pro-regenerative therapeutic option in hard-to-treat patients where standard therapy with N-acetylcysteine alone falls short. Notably, the benefit of focused short-term application of IL-11 or IL-22 in acute disorders, such as APAP-induced ALI, should likely outweigh the inherent danger of these cytokines to promote in the long run tumor growth (74, 86, 118), which has been detected for IL-22 and hepatocellular carcinoma patients (118–120).

## Author Contributions

The author confirms being the sole contributor of this work and approved it for publication.

## Conflict of Interest Statement

The author declares that the research was conducted in the absence of any commercial or financial relationships that could be construed as a potential conflict of interest.
